# Supplementary material for: HPC-Atlas: Computationally Constructing A Comprehensive Atlas of Human Protein Complexes
Source: Genomics Proteomics Bioinformatics. 2023 Sep 18;21(5):976–90. doi: 10.1016/j.gpb.2023.05.001 (PMC10928439; doi:10.1016/j.gpb.2023.05.001)
Supplement: Supplementary File S1 — Algorithm for iteratively adding and removing nodes to generate a protein complex [file mmc1.docx]

**File S1 Algorithm for iteratively adding and removing nodes to generate a protein complex**

| **Algorithm 1 Iteratively generating a protein complex** |
| --- |
| **Input:** The LE-PIN $G=(V,E,W)$,  ${SG}_{v}$ is the subgraph that contains a single protein, from line 12 of article algorithm 1  **Output:** A protein complex, ${SG}_{v}$   1. **Initialize:** $k=1$ /* Record the number of iterations */ 2. **Repeat** 3. **Step 1:** Add the neighbor nodes of the subgraph (${SG}_{v}$) to maximize the value of $F_{SG(v)}$ according to Equation 28 4. **Initialize:** ${NSG}_{(v)}=\{\}$ /* The neighbor node set of the ${SG}_{v}$ */ 5. Insert all neighbor nodes into ${NSG}_{(v)}$ 6. **if** $\left\vert V_{{SG}_{v}} \right\vert=1$ **then** 7. $F_{SG(v)}=0$ and $Expectation\_edges=0$ 8. **else** 9. Calculate the value of $F_{SG(v)}$ and $Expectation\_edges$ for ${SG}_{(v)}$ according to Equations 28 and 30 10. **end if** 11. **while** $\left\vert{NSG}_{(v)} \right\vert\neq0$ **do** 12. **Initialize:** $node\_max=\emptyset$ 13. **for** each protein $n_{s}$ in ${NSG}_{(v)}$ **do** 14. **if** $W(n_{s},V_{{SG}_{v}})>$ 0.5 **then** /* The edge weight of $n_{s}$ with the internal nodes of the ${SG}_{v}$ is greater than 0.5 */ 15. Calculate the value of $F_{SG\left( v \right)+n_{s}}$for the subgraph (${SG}_{v}+n_{s}$) according to Equation 28 16. Calculate the value of $Actually\_edges$ for the subgraph (${SG}_{v}+n_{s}$) according to Equation 31 17. **if** $F_{SG\left( v \right)+n_{s}}> F_{SG(v)}$ and $Actually\_edges\geq Expectation\_edges$ **then** 18. $node\_max=n_{s}$; $F_{SG(v)}=F_{SG\left( v \right)+n_{s}}$; 19. $Expectation\_edges = Actually\_edges$ 20. **end if** 21. **end if** 22. **end for** 23. **if** $node\_max\neq\emptyset$ **then** 24. Add $node\_max$ to ${SG}_{v}$; Remove $node\_max$ from ${NSG}_{(v)}$ 25. **else** 26. break 27. **end if** 28. **end while** 29. **Step 2:** Remove any of inner nodes of the ${SG}_{v}$ to maximize the value of $F_{SG(v)}$ 30. **Initialize:** ${BSG}_{v}=\{\}$ /* The boundary node set of the ${SG}_{v}$ */ 31. **for** each protein $j$ in ${SG}_{v}$ **do** 32. Obtain the neighbor node set of $j$, denote as $Neighbor(j)$ 33. $Interaction\_set = Neighbor(j)\cap V_{{SG}_{v}}$ 34. **if**$Neighbor\left( j \right)-Interaction\_set\neq\emptyset$ **then** /* The difference set among $Neighbor(j)$ and $Interaction\_set$ */ 35. Add $j$ into ${BSG}_{v}$ 36. **end if** 37. **end for** 38. **while** $\left\vert{BSG}_{(v)} \right\vert\neq0$ **do** 39. **initialize:** $node\_min=\emptyset$ 40. **for** each protein $n_{s}$ in ${BSG}_{(v)}$ **do** 41. Calculate the value of $F_{SG\left( v \right)-n_{s}}$ for the subgraph (${SG}_{v}-n_{s}$) according to Equation 28 42. Calculate the value of $Actually\_edges$ for the subgraph (${SG}_{v}-n_{s}$) according to Equation 31 43. **if** $F_{SG\left( v \right)-n_{s}}> F_{SG(v)}$ and $Actually\_edges\geq Expectation\_edges$ **then** 44. $node\_min=n_{s}$; $F_{SG(v)}=$ $F_{SG\left( v \right)-n_{s}}$; 45. $Expectation\_edges = Actually\_edges$ 46. **end if** 47. **end for** 48. **if** $node\_min\neq\emptyset$ **then** 49. Remove $node\_min$ from ${SG}_{v}$; Remove $node\_min$ from ${BSG}_{(v)}$ 50. **else** 51. break 52. **end if** 53. **end while** 54. $k=k+1$ 55. **until** ${SG}_{v}$ is not changing 56. **return** Output the predicted protein complex, ${SG}_{v}$ |
