# Supplementary figures and images for: HPC-Atlas: Computationally Constructing A Comprehensive Atlas of Human Protein Complexes
Source: Genomics Proteomics Bioinformatics. 2023 Sep 18;21(5):976–90. doi: 10.1016/j.gpb.2023.05.001 (PMC10928439; doi:10.1016/j.gpb.2023.05.001)

## Slide 1
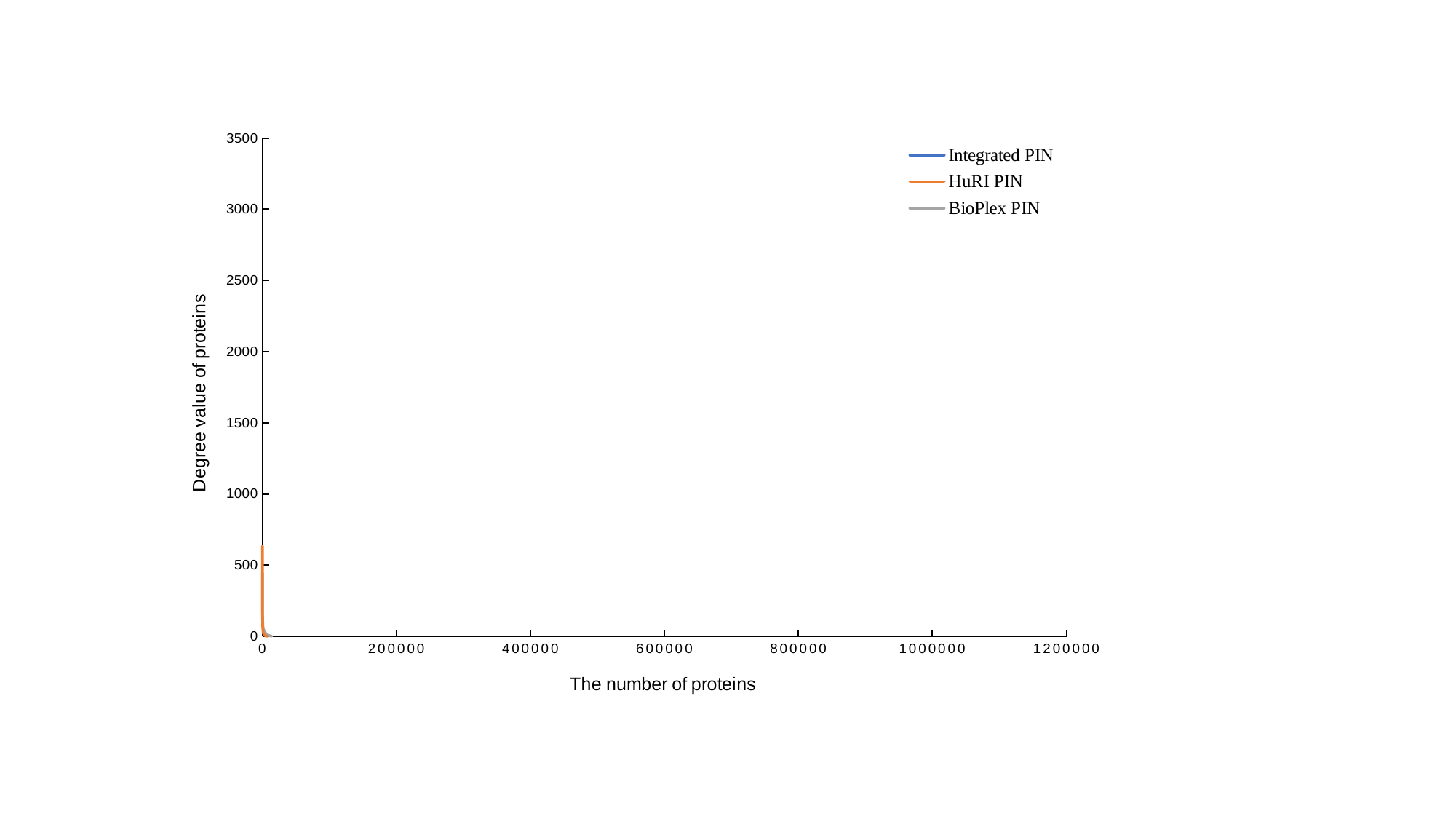

### Chart
| Category | | | |
|---|---|---|---|

Supplement: Supplementary Figure S1 — Degree distribution of proteins in different PINs HuRI, BioPlex, and integrated PIN are similar to scale-free networks. [file mmc2.pptx]

## Slide 1
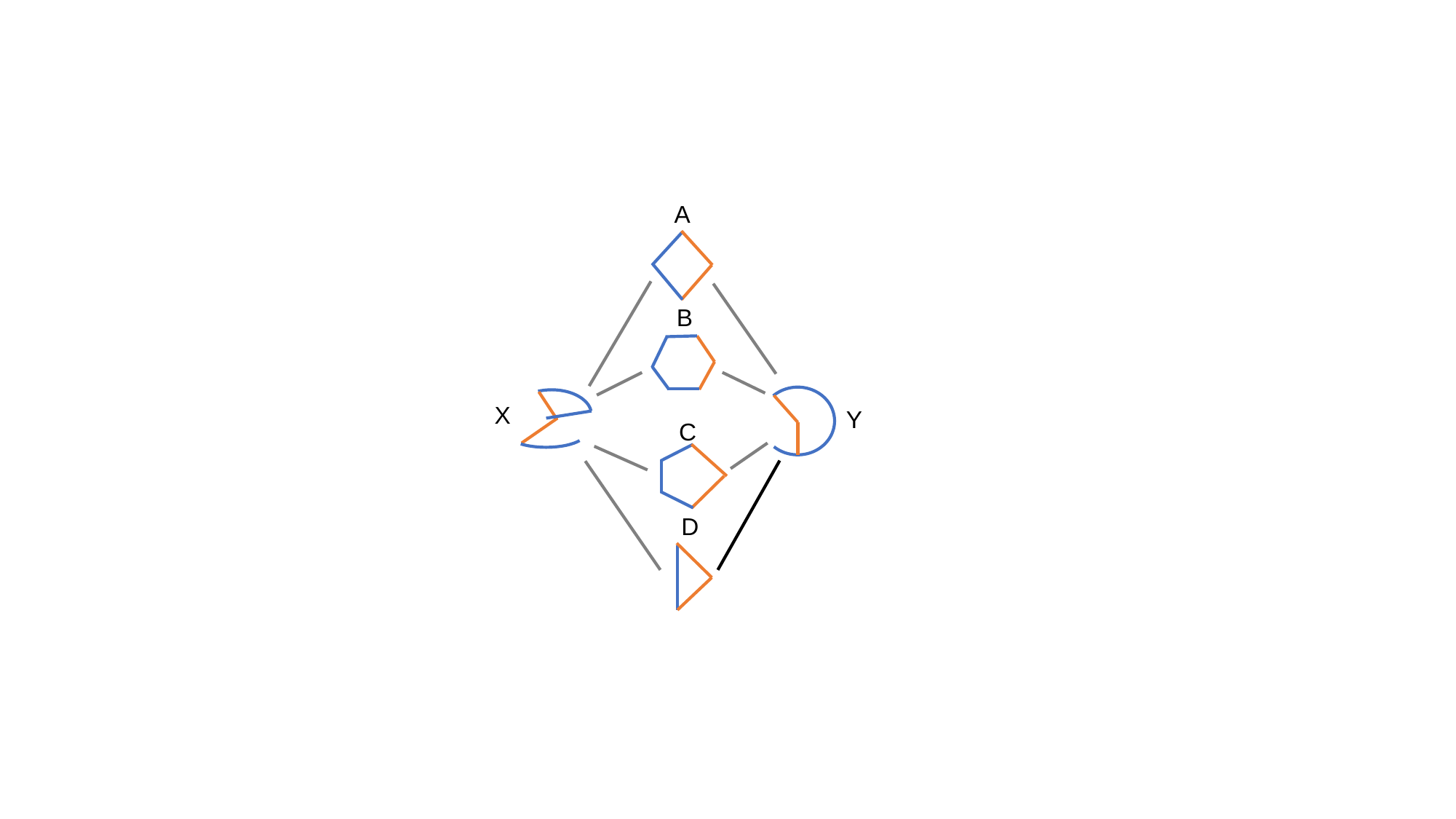

A
B
X
Y
C
D

Supplement: Supplementary Figure S3 — The L3 method diagram PPIs often require complementary interfaces (e.g., protein C and protein Y). And protein D may interact with protein Y (black link), which can be predicted by using L3 method. [file mmc3.pptx]
