# Supplementary material for: HPC-Atlas: Computationally Constructing A Comprehensive Atlas of Human Protein Complexes
Source: Genomics Proteomics Bioinformatics. 2023 Sep 18;21(5):976–90. doi: 10.1016/j.gpb.2023.05.001 (PMC10928439; doi:10.1016/j.gpb.2023.05.001)
Supplement: Supplementary Table S1 — The statistics of PINs [file mmc4.docx]

**Table S1 The statistics of PINs**

| **PIN** | **No. of proteins** | **No. of PPIs** |
| --- | --- | --- |
| HuRI | 8985 | 63,132 |
| BioPlex | 14,484 | 167,932 |
| Integrated PIN | 16,632 | 2,658,160 |

*Note*: HuRI, Human Reference Interactome; PPIs, protein–protein interactions.
