# Supplementary material for: HPC-Atlas: Computationally Constructing A Comprehensive Atlas of Human Protein Complexes
Source: Genomics Proteomics Bioinformatics. 2023 Sep 18;21(5):976–90. doi: 10.1016/j.gpb.2023.05.001 (PMC10928439; doi:10.1016/j.gpb.2023.05.001)
Supplement: Supplementary Table S2 — The statistics of protein complexes sets [file mmc5.docx]

**Table S2 The statistics of protein complexes sets**

| **Protein complexes sets** | **No. of proteins** | **No. of complexes** |
| --- | --- | --- |
| Train set (for classifying edges) | 1352 | 586 |
| Test set (for classifying edges) | 1346 | 591 |
| CORUM 2.0 (for identifying complexes) | 1736 | 1362 |
| Independent set (for novel complexes) | 897 | 518 |

*Note*: CORUM, the comprehensive resource of mammalian protein complexes.
